# The effects of housing stability on service use among homeless adults with mental illness in a randomized controlled trial of housing first

**DOI:** 10.1186/s12913-018-3028-7

**Published:** 2018-03-20

**Authors:** Nick Kerman, John Sylvestre, Tim Aubry, Jino Distasio

**Affiliations:** 1 School of Psychology, University of Ottawa, Ottawa, ON K1N 6N5 Canada; 2 Department of Geography, University of Winnipeg, Winnipeg, MB R3B 2E9 Canada

**Keywords:** Homelessness, Housing first, Mental disorders, Service use

## Abstract

**Background:**

Housing First is an effective intervention to stably house and alter service use patterns in a large proportion of homeless people with mental illness. However, it is unknown whether there are differences in the patterns of service use over time among those who do or do not become stably housed and what effect, if any, Housing First has on these differing service use patterns. This study explored changes in the service use of people with mental illness who received Housing First compared to standard care, and how patterns of use differed among people who did and did not become stably housed.

**Methods:**

The study design was a multi-site randomized controlled trial of Housing First, a supported housing intervention. 2039 participants (Housing First: *n* = 1131; standard care: *n* = 908) were included in this study. Outcome variables include nine types of self-reported service use over 24 months. Linear mixed models examined what effects the intervention and housing stability had on service use.

**Results:**

Participants who achieved housing stability, across the two groups, had decreased use of inpatient psychiatric hospitals and increased use of food banks. Within the Housing First group, unstably housed participants spent more time in prison over the study period. The Housing First and standard care groups both had decreased use of emergency departments and homeless shelters.

**Conclusions:**

The temporal service use changes that occurred as homeless people with mental illness became stably housed are similar for those receiving Housing First or standard care, with the exception of time in prison. Service use patterns, particularly with regard to psychiatric hospitalizations and time in prison, may signify persons who are at-risk of recurrent homelessness. Housing support teams should be alert to the impacts of stay-based services, such as hospitalizations and incarcerations, on housing stability and offer an increased level of support to tenants during critical periods, such as discharges.

**Trial registration:**

ISRCTN. ISRCTN42520374. Registered 18 August 2009.

## Background

Mental illness is a pervasive problem among people who are chronically homeless. Given the high prevalence of mental health problems, as well as the increased risks of developing medical conditions while homeless, there is frequent use of hospital and crisis services by this population [1–4]. In addition, psychiatric hospitalizations of homeless people are longer and more expensive than for the general population, likely related to their presenting at admission with more severe and complex psychiatric illnesses [5]. The use of these more acute services is often due to barriers accessing ambulatory or specialist services in the community that could suitably address presenting health problems, placing an unnecessary and expensive burden on health systems [6–8].

Housing First is an evidence-based intervention that involves the provision of scattered-site housing with a rental subsidy and accompanying support without any pre-conditions for eligibility (e.g., there are no requirements about abstinence from substance use or existing involvement with mental health services) [9]. Support services are provided through Assertive Community Treatment (ACT) teams or case managers. However, to promote choice, tenants may receive as much or as little support as they choose and may even refuse services all together [10]. Research on the model has demonstrated that Housing First is effective in stably housing a large majority of homeless people with mental illness. Recent studies by Aubry et al. found that 73% and 71% of participants who received Housing First with ACT support were stably housed at 12 and 24 months, respectively [11, 12]. At both time points, the percentage of Housing First participants who were stably housed was significantly greater than those who received standard care. Similarly, 78% of individuals receiving Housing First with Intensive Case Management (ICM) were stably housed for 50% of the time or more between 12 months and 24 months compared to only 39% of the time for a standard care group [13]. These findings are consistent with those from other past studies (e.g., [10, 14, 15]).

The Housing First intervention also affects homeless adults’ use of other services. First, given that Housing First is associated with increased rates of housing stability, it also produces drastic reductions in the use of homeless shelters (e.g., [16, 17]). As for mental health services, Housing First has been shown to change patterns of service use. Reductions in emergency department visits and hospitalizations are the service domains where the evidence is strongest [18, 19]. Less research has focused on the use of outpatient mental health services following housing entry. However, in a quasi-experimental study of Housing First, individuals who received the intervention had significantly greater outpatient mental health service use in all domains (case management, medication management, and therapy/rehabilitation) in the year after becoming housed [20]. The study, which also found reductions in inpatient service use, suggests that Housing First may facilitate more appropriate use of less-intensive mental health resources that better fit the needs of individuals [20]. Lastly, Housing First is known to affect people’s interactions with the criminal justice system, with a recent systematic review finding strong evidence that the intervention is effective in reducing arrests and incarcerations [21].

Overall, Housing First is effective in stably housing a large majority of people with mental illness and reducing burden on service systems through greater uptake of outpatient services and less reliance on acute and institutional services. However, very little is known about the people who do not achieve housing stability via the Housing First model and experience recurrent homelessness. A recent study examined differences among people who do and do not achieve housing stability in the first year of tenancy via Housing First [22]. Findings showed that participants who did not become stably housed were more likely, at baseline, to: have a psychotic disorder, feel more psychologically integrated into their communities, report higher quality of life, and have spent more time in prison in recent months. Although these predictors of housing stability were significant, the strength of association was relatively small for all variables.

From the same study, Adair et al. examined housing trajectories over a 24-month period across treatment groups (i.e., Housing First and standard care participants were merged together) [23]. Findings showed that participants who remained unstably housed had longer histories of homelessness but fewer hospitalizations at baseline than those who became stably housed early on. Further, compared to the unstably housed group, participants who had early success in housing but later lost their housing were more likely to have greater psychiatric symptoms and more past hospitalizations. However, because the studies only examined baseline predictors of housing stability [22, 23], it is unclear how the groups differed once housed. In particular, it is unknown whether individuals who become stably housed display different patterns of service use over time than those who encounter difficulties and what effect, if any, Housing First has on differing service use patterns. This study sought to advance the limited evidence on the characteristics of people who experience difficulties in Housing First by exploring their patterns of service use and comparing them to individuals who become successfully housed by the intervention. A greater understanding of the patterns of service use that are associated with successful community living and ones that may be risk factors for recurrent homelessness will be valuable for determining how Housing First tenants can be better supported.

### Current study

Using 24-month longitudinal data from a randomized controlled trial (RCT) of Housing First conducted in Canada, this study examined two research questions. First, how does service use by people who do and do not become stably housed change over time? It is hypothesized that, as participants become stably housed, they will have fewer hospitalizations and time spent in prison, as well as less use of emergency departments and crisis services, shelters, and drop-in centers. In contrast, use of outpatient hospital services and food banks will increase. Among participants who struggle to become stably housed, or experience difficulties once housed and become recurrently homeless, it is expected that their use of health services, and drop-in centers will remain unchanged, whereas amount of time spent in shelters and prisons will increase.

The second research question is: What impact does Housing First have on the service use patterns of people who do and do not become stably housed? It is hypothesized that participants’ housing stability will have a stronger relationship with changes in service use over 24 months and that Housing First will minimally affect service use patterns.

## Methods

### Design

This study used data from the At Home/Chez Soi demonstration project, a RCT that was conducted in five Canadian cities (Moncton, Montreal, Toronto, Vancouver, and Winnipeg). Participants were randomly assigned to receive either Housing First (with support via an ACT or ICM model) or standard care. Data were collected from October 2009 to June 2013, with each participant being followed for a maximum of 24 months. Participants were recruited from community service organizations, including shelters, drop-in centres, street outreach teams, and health clinics, as well as directly off the street. For more information about the trial design, see the published protocol [24].

### Study participants

Data were obtained from 2255 individuals who met the following trial inclusion conditions: (1) either had a recent diagnosis of a mental illness or met criteria for a current mental disorder, as determined by the Mini International Neuropsychiatric Interview (MINI) [25]; (2) lived in Moncton, Montreal, Toronto, Vancouver, or Winnipeg; (3) were homeless at study entry (defined as having no fixed address, or having used homeless shelters for one or more nights in the previous month); and (4) were 18 years of age or older (19+ in Vancouver). All participants provided written consent.

**Fig. 1:**
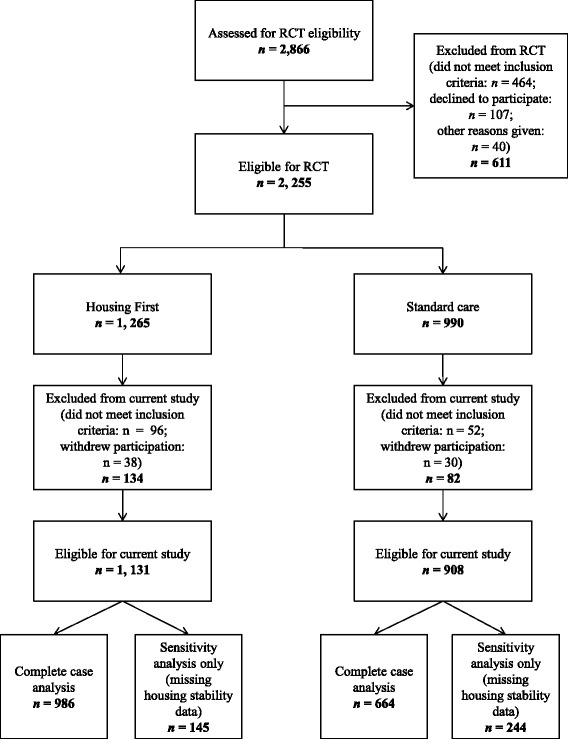
Screening, randomization, and analysis procedures of participants for RCT and current study

Because changes in service use can occur quickly after obtaining housing, participants were also required to be unstably housed at baseline (50% or fewer days in stable housing in previous three months) – the point at which initial data were also collected on service use. Using this criterion, 148 individuals who had spent more than 50% of time in stable housing at their baseline assessments were deemed not to be unstably housed and excluded from this study. An additional 68 individuals withdrew prior to 24 months and were not included in data analysis. The final sample comprised of 2039 participants (see Fig. 1).

### Intervention

#### Housing first

Participants who were randomly assigned to the intervention group received housing and support via the Housing First model. The model includes rent subsidies; choice around housing and support; and apartments via the private rental market, though some individuals were offered housing via other settings (e.g., social housing) [26]. A small number of Housing First participants at the Vancouver site were randomized to a congregate, supportive housing model (more information about the third arm intervention is published elsewhere) [27].

Housing First participants received support services via either ACT teams or ICM. ACT teams included a psychiatrist, nurse, social worker, and peer specialist, as well as other clinicians. Services were available around the clock, seven days per week, and the teams’ staff to participant ratios were 1:10. Individuals with moderate needs received ICM, whereas those with high needs were provided ACT. Participants were determined to have high needs if they met one of the following conditions: a score within the severe or medium disability range on the Multnomah Community Ability Scale (MCAS) [28], a MINI diagnosis of a psychotic or bipolar disorder, a comorbid substance use disorder, two or more hospitalizations within a single year during the past five years, or involvement with the criminal justice system within the previous six months. ICM involved teams of case managers with staff to participant ratios that did not exceed 1:16 and the provision of services 12 h a day, seven days a week.

#### Standard care

Participants who received standard care had access to all of the existing housing and support services offered in their communities other than from the Housing First programs. As the trial took place in five cities across Canada, the programs available to participants in this group likely differed as a function of their location.

### Measures

Assessments comprised of questionnaires that were completed in an interview format with participants every three or six months over a 24-month period. This study primarily used data collected from two scales: Health, Social, and Justice Service Use Inventory [29]; and Residential Time-line Follow-back [30]. The former was administered every six months, whereas the latter was completed every three months. In addition, data from the Demographics, Service, and Housing History [29]; MCAS [28]; and Global Appraisal of Individual Needs–Substance Problem Scale (GAIN-SPS) [31] were used to describe the sample at baseline. Details about how these three measures were used in the trial can be found in the published protocol [24].

The Health, Social, and Justice Service Use Inventory (HSJSU) [24] is a self-report questionnaire that was used to assess use of health, social, and justice services in the previous six months. Within these service domains, data are collected on volume of use, name of service, and purpose of use. For this study, data on volume of use of the following types of services were analyzed: outpatient hospital programs, overnight hospital stays (non-emergency department, and not including laboratory or diagnostic tests), emergency departments, crisis lines, crisis teams, drop-in centers, and food banks. Use of justice services, as assessed by the HSJSU, were not examined in this study. The HSJSU was developed for the RCT and it previously underwent pre-testing and piloting to ensure that individuals with serious mental illness do not experience difficulties with the recall items [24]. Also, its accuracy was tested at the Vancouver site by comparing self-reported service use on the measure to service use from an administrative database. There was moderate to almost perfect correspondence between the data for psychiatric hospitalizations, emergency department visits, and time spent in prison [32].

The Residential Time-line Follow-back (RTLFB) [30] was used to assess housing histories in the previous three months, with the exception of the initial assessment, which occurred at the three-month time point and examined the previous six months (i.e., three months before and after baseline). The RTLFB collects information on each type of residence lived in during that period and the number of days spent there. The residences are then categorized as either a street place, stable residence, temporary or unstable residence, emergency or street crisis location, or institution. This study used data from two categorizations: stable residences and institutions. Stable residences were defined as stays in any of the following locations: own single room occupancy hotels, own apartment or house, apartment or house of a family member or of someone else for an intended stay duration of six or more months, boarding house, transitional housing program for an intended stay duration of six or more months, or a group home [30]. Use of several institutional services assessed by the RTLFB were examined as part of this study. These included days spent in hospital (i.e., overnight, non-emergency department; psychiatric hospital, general hospital for psychiatric purposes, and general hospital for medical purposes), homeless shelters, and prison. The RTLFB was developed and validated for use with the homeless population. It has adequate two-week, test-retest reliability; has adequate concurrent validity, as assessed through correlations between housing agency and self-reports; and is sensitive to change in residential stability [30].

### Data analysis

A series of linear mixed models were conducted that each had three fixed factors – (1) time, (2) intervention, and (3) housing stability. The fixed factor of time had three levels (baseline, 12 months, and 24 months) and represented the points at which service use data are analyzed. Intervention comprised of two groups (Housing First or standard care). No comparisons were made between Housing First participants who received ACT and those who received ICM. The third factor, housing stability, comprised of four groups: sustained housing stability, late housing stability, sustained housing instability, and late housing instability. Housing stability was computed using modified procedures by Volk and colleagues [22]. Following baseline, housing stability was determined by the proportion of time spent in stable housing accommodations over 12 months. More than 50% was considered to be stably housed. However, if participants had spent 100% of their time in stable housing in the previous 3 months, they were categorized as stably housed, regardless of their housing accommodations in the prior nine months. These procedures produced indicators of housing stability at 12 months and 24 months. Participants who were stably housed at both 12 and 24 months were determined to have achieved sustained housing stability, whereas individuals who were unstably housed at those two time points were classified as having sustained housing instability. Late housing stability participants are those who were initially unstably housed at 12 months but stably housed by 24 months. In contrast, late housing instability refers to participants who were stably housed at 12 months but became unstably housed by 24 months.

The dependent variables in the mixed models were nine unique types of service use. The types of service use were: (1) emergency departments (visits); (2) overnight hospital stays for medical reasons (days); (3) overnight hospital stays for psychiatric reasons (days); (4) outpatient hospital programs (visits); (5) specialized crisis services (calls to crisis lines and visits by crisis teams); (6) drop-in centers (visits); (7) homeless shelters (days); (8) food banks (visits); and (9) prisons (days). Because service use was assessed in six-month intervals, to generate values that were comparable to baseline, scores at 12 and 24 months were mean ratings of the previous year (i.e., combined data from two time points). To balance statistical error rates, Bonferroni corrections were applied to all pairwise comparisons within each linear mixed model, as opposed to adjustments across the total number of statistical tests. Using a software-adjusted Bonferroni computation, alphas ≤ .05 were considered to be significant. To measure effect sizes, adjusted standardized mean differences were computed for all significant pairwise comparisons and two-level main effects following procedures by Borenstein et al. [33].

Missing data were generally low, ranging from 3 to 15%; however, when determinations of overall housing stability were merged across time points, 389 (19.1%) participants were missing data on the independent variable. To evaluate the effects of the missing data, a sensitivity analysis was performed using 20 multiply imputed datasets that had a predictive mean matching algorithm. Following guidelines by Graham [34] to reduce bias in multiple imputation procedures, models comprised of 85 variables involved in the study’s analyses and an additional 50 auxiliary predictor variables. Data augmentation involved a total of 4000 steps (200 iterations per imputation). Consistency between the complete case analysis and pooled multiple imputation results was assessed by measuring the degree of overlap in the estimated confidence intervals between the two analyses. Where results from the complete case analysis were consistent with the multiple imputation analysis, only the former are presented. Where results from the two analyses deviated, findings from both are discussed. All statistical analyses were performed using SPSS 24.

## Results

The characteristics of the sample at baseline are displayed in Table 1. Adjusted mean ratings of use for the nine service domains by intervention condition and housing stability are listed in Table 2. No significant differences in characteristics or service use at baseline were found between the Housing First and standard care groups.

**Table 1:** Baseline characteristics by intervention and housing stability

Characteristic	Full Sample* (*N* = 1581–1650)	Housing First				Standard Care			
		Sustained Housing Instability (*n* = 85–87)	Late Housing Instability (*n* = 84–89)	Sustained Housing Stability (*n* = 708–732)	Late Housing Stability (*n* = 71–78)	Sustained Housing Instability (*n* = 296–312)	Late Housing Instability (*n* = 32–34)	Sustained Housing Stability (*n* = 153–158)	Late Housing Stability (*n* = 152–160)
Age	41.03 (11.06)	38.07 (11.87)	39.53 (11.12)	41.49 (11.01)	39.34 (9.96)	41.63 (11.13)	39.18 (9.81)	41.51 (11.58)	40.94 (10.69)
Gender, male	1127 (68.3%)	65 (74.7%)	72 (80.9%)	479 (65.4%)	52 (66.7%)	226 (72.4%)	25 (73.5%)	103 (65.2%)	105 (65.6%)
Canadian-born	1334 (80.8%)	72 (82.8%)	77 (86.5%)	584 (79.8%)	66 (84.6%)	255 (81.7%)	28 (82.4%)	125 (79.1%)	127 (79.4%)
Lifetime length of homelessness (months)	61.60 (70.56)	84.55 (85.81)	80.45 (95.26)	55.74 (65.33)	68.74 (71.83)	64.98 (69.80)	66.85 (73.64)	55.60 (71.51)	60.52 (63.97)
Military veteran	70 (4.3%)	4 (4.7%)	3 (3.4%)	35 (4.8%)	4 (5.1%)	14 (4.5%)	2 (5.9%)	5 (3.2%)	3 (1.9%)
MCAS total	59.70 (8.65)	57.60 (8.80)	58.62 (8.33)	60.13 (8.69)	58.18 (9.79)	58.61 (8.72)	59.32 (9.02)	61.82 (7.93)	60.32 (7.93)
GAIN-SPS total (past month)	1.84 (1.94)	2.08 (2.07)	2.42 (2.06)	1.71 (1.91)	1.97 (2.07)	2.07 (1.96)	2.09 (2.02)	1.53 (1.85)	1.68 (1.82)
Chronic medical conditions	4.69 (3.43)	3.95 (3.18)	5.51 (3.63)	4.70 (3.42)	4.01 (3.32)	4.63 (3.45)	5.03 (4.00)	4.50 (3.23)	5.16 (3.49)
Current psychiatric diagnosis									
Major depressive episode	839 (50.8%)	40 (46.0%)	49 (55.1%)	381 (52.0%)	35 (44.9%)	142 (45.5%)	19 (55.9%)	83 (52.5%)	90 (56.3%)
Mania or hypomania episode	221 (13.4%)	9 (10.3%)	14 (15.7%)	104 (14.2%)	6 (7.7%)	47 (15.1%)	4 (11.8%)	20 (12.7%)	17 (10.6%)
Posttraumatic stress disorder	476 (28.9%)	21 (24.1%)	37 (41.6%)	216 (29.5%)	13 (16.7%)	82 (26.4%)	11 (32.4%)	50 (31.6%)	46 (28.8%)
Panic disorder	375 (22.7%)	11 (12.6%)	24 (27.0%)	171 (23.4%)	12 (15.4%)	81 (26.0%)	8 (23.5%)	31 (19.6%)	37 (23.1%)
Mood disorder, psychotic features	276 (16.7%)	13 (14.9%)	11 (12.4%)	123 (16.8%)	10 (12.8%)	55 (17.7%)	4 (11.8%)	32 (20.3%)	28 (17.5%)
Psychotic disorder	599 (36.3%)	38 (43.7%)	23 (25.8%)	254 (34.7%)	35 (44.9%)	127 (40.7%)	11 (32.4%)	54 (34.2%)	57 (35.6%)
Alcohol abuse/dependence	740 (44.8%)	41 (47.1%)	57 (64.0%)	311 (42.5%)	33 (42.3%)	151 (48.4%)	12 (35.3%)	63 (39.9%)	72 (45.0%)
Drug abuse/dependence	884 (53.6%)	50 (57.5%)	56 (62.9%)	371 (50.7%)	44 (56.4%)	174 (55.8%)	24 (70.6%)	78 (49.4%)	87 (54.4%)
Long-stay psychiatric hospitalization (≥6 months) in past 5 years	109 (6.7%)	9 (10.8%)	5 (5.7%)	53 (7.3%)	6 (7.8%)	14 (4.5%)	1 (3.0%)	11 (7.1%)	10 (6.3%)
Two or more psychiatric hospitalizations in past 5 years	602 (37.2%)	18 (21.2%)	26 (29.2%)	272 (37.9%)	30 (40.0%)	113 (37.0%)	12 (36.4%)	60 (38.2%)	71 (44.9%)
Support model									
ACT	342 (34.7%)	34 (39.1%)	33 (37.1%)	246 (33.6%)	29 (37.2%)	–	–	–	–
ICM	560 (56.8%)	50 (57.4%)	52 (58.4%)	417 (57.0%)	41 (52.6%)	–	–	–	–
Congregate, supportive housing	84 (8.5%)	3 (3.4%)	4 (44.9%)	69 (9.4%)	8 (10.3%)	–	–	–	–

*ACT* Assertive Community Treatment, *ICM* Intensive Case Management, *MCAS* Multnomah Community Ability Scale, *GAIN-SPS* Global Appraisal of Individual Needs–Substance Problem Scale

*Full sample for support model includes only Housing First participants (*N* = 986)

*Notes*. Higher scores on the MCAS reflect greater functioning. Higher scores on the GAIN-SPS reflect greater severity of substance problems

**Table 2:** Adjusted service use means (95% confidence intervals) across 24 months by intervention and housing stability

Service Use Domain	Housing First				Standard Care			
	Sustained Housing Instability (*n* = 82–87)	Late Housing Instability (*n* = 87–89)	Sustained Housing Stability (*n* = 712–732)	Late Housing Stability (*n* = 72–78)	Sustained Housing Instability (*n* = 299–312)	Late Housing Instability (*n* = 32–34)	Sustained Housing Stability (*n* = 153–158)	Late Housing Stability (*n* = 155–160)
Emergency department (visits/6 months)								
Baseline	2.30 (1.51, 3.09)	3.55 (2.78, 4.33)	1.92 (1.66, 2.19)	1.92 (1.08, 2.76)	1.71 (1.30, 2.12)	2.68 (1.44, 3.91)	2.05 (1.47, 2.63)	2.11 (1.54, 2.69)
Year 1	0.61 (− 0.18, 1.40)	2.03 (1.27, 2.80)	1.05 (0.78, 1.31)	1.08 (0.26, 1.90)	1.33 (0.91, 1.75)	1.32 (0.06, 2.57)	1.11 (0.53, 1.68)	1.97 (1.39, 2.54)
Year 2	0.59 (− 0.20, 1.38)	1.50 (0.73, 2.27)	0.83 (0.56, 1.10)	0.74 (− 0.07, 1.56)	1.14 (0.73, 1.55)	1.10 (−0.13, 2.34)	0.83 (0.25, 1.41)	1.04 (0.47, 1.62)
Hospital stays, medical (days/3 months)								
Baseline	0.13 (− 0.83, 1.08)	0.37 (− 0.58, 1.32)	0.94 (0.61, 1.27)	0 (−1.01, 1.01)	0.54 (0.04, 1.05)	0 (−1.53, 1.53)	0.50 (−0.21, 1.21)	1.51 (0.81, 2.22)
Year 1	0.17 (−0.79, 1.13)	0.33 (−0.62, 1.28)	0.32 (− 0.01, 0.65)	1.12 (0.11, 2.13)	0.68 (0.17, 1.18)	0.04 (− 1.50, 1.57)	0.25 (− 0.46, 0.96)	0.46 (− 0.25, 1.17)
Year 2	0.49 (− 0.46, 1.45)	0.55 (− 0.40, 1.49)	0.53 (0.20, 0.86)	0.51 (− 0.50, 1.52)	1.09 (0.58, 1.59)	0.15 (− 1.38, 1.69)	0.49 (− 0.22, 1.20)	0.44 (− 0.27, 1.15)
Hospital stays, psychiatric (days/3 months)								
Baseline	1.28 (− 0.98, 3.53)	1.78 (− 0.45, 4.00)	4.22 (3.44, 4.99)	4.92 (2.55, 7.30)	2.67 (1.48, 3.86)	2.00 (− 1.60, 5.60)	3.95 (2.28, 5.60)	3.80 (2.14, 5.46)
Year 1	2.98 (0.73, 5.23)	2.01 (−0.22, 4.24)	1.54 (0.77, 2.32)	5.16 (2.78, 7.54)	3.30 (2.11, 4.49)	0.39 (−3.20, 3.99)	0.84 (−0.83, 2.51)	3.00 (1.34, 4.66)
Year 2	4.56 (2.31, 6.82)	3.11 (0.88, 5.34)	0.92 (0.14, 1.69)	2.24 (−0.14, 4.62)	2.97 (1.79, 4.16)	0.53 (−3.07, 4.13)	0.54 (−1.13, 2.21)	1.31 (−0.35, 2.97)
Outpatient hospital services (visits/6 months)								
Baseline	0.57 (−0.42, 1.56)	1.15 (0.17, 2.13)	1.54 (1.20, 1.88)	0.46 (−0.62, 1.54)	0.87 (0.35, 1.40)	1.28 (−0.34, 2.90)	1.44 (0.70, 2.18)	1.35 (0.62, 2.09)
Year 1	0.40 (−0.60, 1.41)	0.33 (−0.65, 1.30)	0.86 (0.52, 1.20)	0.41 (−0.64, 1.46)	0.97 (0.44, 1.50)	0.42 (−1.17, 2.02)	2.37 (1.64, 3.10)	1.01 (0.28, 1.75)
Year 2	0.42 (−0.59, 1.42)	0.17 (−0.81, 1.15)	0.59 (0.25, 0.94)	0.56 (−0.48, 1.60)	0.83 (0.31, 1.35)	3.41 (1.84, 4.99)	1.56 (0.82, 2.30)	0.95 (0.22, 1.68)
Specialized crisis services (calls and visits/6 months)								
Baseline	2.00 (0.51, 3.49)	1.29 (−0.16, 2.75)	1.36 (0.85, 1.87)	0.97 (−0.61, 2.56)	0.77 (−0.01, 1.56)	1.59 (− 0.77, 3.94)	1.68 (0.58, 2.79)	1.19 (0.09, 2.29)
Year 1	0.39 (−1.12, 1.90)	1.13 (−0.33, 2.58)	0.75 (0.25, 1.26)	0.91 (−0.66, 2.47)	0.52 (−0.27, 1.32)	0.62 (−1.77, 3.01)	1.61 (0.52, 2.70)	0.85 (−0.25, 1.95)
Year 2	0.46 (−1.03, 1.96)	0.48 (−0.99, 1.94)	1.45 (0.94, 1.96)	0.62 (−0.93, 2.18)	0.66 (−0.12, 1.44)	0.43 (−1.93, 2.78)	0.93 (− 0.18, 2.03)	1.53 (0.44, 2.62)
Drop-in centers (visits/6 months)								
Baseline	75.30 (55.64, 94.96)	71.78 (52.45, 91.10)	68.26 (61.51, 75.00)	86.77 65.72, 107.83)	86.82 (76.40, 97.24)	45.39 (13.66, 77.13)	63.34 (48.74, 77.94)	84.59 (70.18, 99.00)
Year 1	62.63 (42.62, 82.64)	63.92 (44.59, 83.24)	47.59 (40.85, 54.33)	54.96 (34.18, 75.73)	88.65 (78.10, 99.19)	54.24 (22.51, 85.98)	36.87 (22.37, 51.38)	71.72 (57.08, 86.36)
Year 2	70.43 (50.54, 90.32)	73.70 (54.27, 93.13)	40.88 (34.08, 47.69)	53.22 (32.58, 73.86)	68.47 (58.14, 78.81)	54.13 (22.87, 85.40)	29.07 (14.42, 43.71)	58.79 (44.29, 73.30)
Homeless shelters (days/3 months)								
Baseline	32.31 (27.02, 37.59)	24.78 (19.55, 30.00)	32.17 (30.35, 33.99)	33.23 (27.65, 38.80)	31.13 (28.34, 33.91)	32.72 (24.28, 41.17)	34.91 (30.99, 38.83)	31.39 (27.50, 35.29)
Year 1	18.68 (13.40, 23.97)	5.55 (0.33, 10.77)	5.31 (3.49, 7.13)	16.56 (10.98, 22.14)	23.63 (20.84, 26.42)	12.05 (3.61, 20.50)	10.12 (6.20, 14.04)	22.93 (19.04, 26.83)
Year 2	14.31 (9.02, 19.59)	6.46 (1.24, 11.68)	0.85 (−0.98, 2.67)	3.61 (−1.97, 9.19)	16.92 (14.13, 19.71)	9.61 (1.16, 18.06)	0.82 (−3.10, 4.74)	6.01 (2.11, 9.90)
Food banks (visits/6 months)								
Baseline	1.29 (0.28, 2.30)	1.82 (0.82, 2.82)	1.95 (1.60, 2.29)	1.49 (0.41, 2.56)	1.97 (1.44, 2.51)	0.74 (−0.87, 2.34)	2.29 (1.54, 3.03)	1.41 (0.67, 2.15)
Year 1	0.85 (−0.18, 1.88)	2.16 (1.17, 3.15)	3.32 (2.97, 3.66)	1.21 (0.15, 2.28)	1.70 (1.15, 2.24)	1.86 (0.23, 3.49)	2.83 (2.08, 3.57)	2.08 (1.33, 2.83)
Year 2	0.85 (−0.18, 1.87)	1.55 (0.55, 2.55)	3.14 (2.79, 3.49)	2.64 (1.58, 3.70)	1.58 (1.05, 2.11)	2.82 (1.22, 4.43)	2.54 (1.79, 3.29)	2.84 (2.10, 3.59)
Prison (days/3 months)								
Baseline	9.00 (6.51, 11.49)	4.15 (1.69, 6.60)	1.42 (0.56, 2.28)	6.50 (3.87, 9.13)	4.19 (2.88, 5.51)	3.09 (−0.89, 7.07)	1.63 (− 0.22, 3.47)	1.19 (− 0.64, 3.03)
Year 1	17.78 (15.29, 20.26)	2.08 (−0.38, 4.54)	0.79 (− 0.06, 1.65)	8.83 (6.21, 11.46)	5.32 (4.01, 6.63)	0.70 (−3.28, 4.68)	0.77 (−1.07, 2.62)	0.64 (−1.19, 2.48)
Year 2	22.72 (20.24, 25.21)	12.25 (9.79, 14.71)	0.82 (−0.04, 1.67)	3.18 (0.55, 5.81)	6.89 (5.58, 8.21)	4.71 (0.74, 8.69)	0.40 (−1.44, 2.25)	0.45 (−1.38, 2.28)

### Use of health services

Visits to the emergency department in the previous six months declined over time for all groups (*p* < .001). A significant decrease occurred from baseline to 12 months (adjusted standardized mean difference [ASMD] = 0.15, *p* < .001, 95% CI = 0.08–0.22), and this change from baseline was maintained at 24 months (ASMD = 0.21, *p* < .001, 95% CI = 0.13–0.28). A significant main effect was also found for housing stability (*p* = .01). Follow-up comparisons showed that late housing instability participants had greater use of emergency departments across the two-year study period than those who experienced sustained housing instability (ASMD = 0.12, *p* = .02, 95% CI = 0.01–0.23) or sustained housing stability (ASMD = 0.11, *p* = .01, 95% CI = 0.02–0.20). No differences were found by intervention. As for use of specialized crisis services in the previous six months, no changes were observed across time for any group.

Findings showed a significant interaction between time and housing stability (*p* < .001) for days spent in hospital for psychiatric reasons in the previous three months. Sustained stably housed participants had a significant decrease in their psychiatric hospital stays from baseline to 12 months (ASMD = 0.16, *p* < .001, 95% CI = 0.07–0.24). This change from baseline was maintained at 24 months (ASMD = 0.23, *p* < .001, 95% CI = 0.12–0.33). Late stably housed participants also had decreased use of psychiatric hospital services from baseline to 24 months (ASMD = 0.21, *p* = .04, 95% CI = .01–0.41). No changes were observed among sustained or late housing instability participants. As for medical hospitalizations in the previous three months, no significant changes were found over the two-year period for any group.

Use of outpatient hospital services in the previous six months was generally low across the study period, with less than two visits on average for most groups (see Table 2). Participants in the standard care condition had higher use of outpatient services than did those in the Housing First condition (*p* < .001; pairwise comparison: ASMD = 0.08, *p* < .001, 95% CI = 0.04–0.11). Further, there was a main effect of housing stability (*p* < .01), with follow-up comparisons showing that sustained housing stability participants had higher usage of outpatient hospital services than individuals who experienced sustained housing instability (ASMD = 0.08, *p* < .01, 95% CI = 0.02–0.15) or late housing instability (ASMD = 0.07, *p* = .04, 95% CI = 0–0.14). No significant changes were found for any group over time.

### Use of community services

There was a significant interaction between time and housing stability for use of homeless shelters in the previous three months (*p* < .001). All groups displayed decreased use at 12 months (sustained housing stability: ASMD = 0.56, *p* < .001, 95% CI = 0.48–0.64; late housing stability: ASMD = 0.32, *p* < .001, 95% CI = 0.17–0.47; sustained housing instability: ASMD = 0.22, *p* < .001, 95% CI = 0.11–0.33; late housing instability: ASMD = 0.56, *p* < .001, 95% CI = 0.32–0.79). This continued to significantly decrease further from 12 to 24 months for the sustained housing stability (ASMD = 0.20, *p* < .001, 95% CI = 0.09–0.30), late housing stability (ASMD = 0.45, *p* < .001, 95% CI = 0.27–0.62), and sustained housing instability groups (ASMD = 0.11, *p* < .001, 95% CI = 0.01–0.20). Only late housing instability participants showed no further change.

There was also a main effect of intervention on use of homeless shelters (*p* < .01), with standard care participants having greater use of homeless shelters than the Housing First group (ASMD = 0.06, 95% CI = 0.02–0.10). However, the estimated parameters of the complete case analysis for standard care participants (*M* = 19.35, SE = 0.77, 95% CI = 17.84–20.86) differed greatly from that of the sensitivity analysis (*M* = 17.64, SE = 0.67, 95% CI = 16.32–18.96). Given the directionality of change in the mean, caution is needed in the interpretation of this main effect.

Visits to drop-in centers in the previous six months declined over time for all groups (*p* = .001). Pairwise comparisons for the full sample showed a significant decrease from baseline to 24 months (ASMD = 0.07, *p* = .03, 95% CI = 0.01–0.13). Use of drop-in centers also significantly differed as a function of housing stability and intervention (*p* = .02). Follow-up pairwise comparisons showed that, among sustained stably housed participants, use was higher for those who received Housing First compared to standard care (ASMD = 0.07, *p* = .05, 95% CI = 0–0.14).

An interaction effect between time and housing stability was present for use of food banks in the previous six months (*p* = .03). Follow-up analyses showed significant changes among the sustained housing stability (*p* < .01) and late housing stability groups (*p* = .01). In particular, sustained stably housed participants displayed increased use from baseline to 12 months (ASMD = 0.13, *p* < .01, 95% CI = 0.03–0.23). Use of food banks for this group at 24 months remained significantly higher than baseline (ASMD = 0.03, *p* = .01, 95% CI = − 0.05-0.11). As for the late housing stability group, they also showed an increase in their use of food banks but this was more gradual over time, with a significant change only occurring between baseline to 24 months (ASMD = 0.18, *p* < .01, 95% CI = 0–0.36).

### Use of prisons

**Fig. 2:**
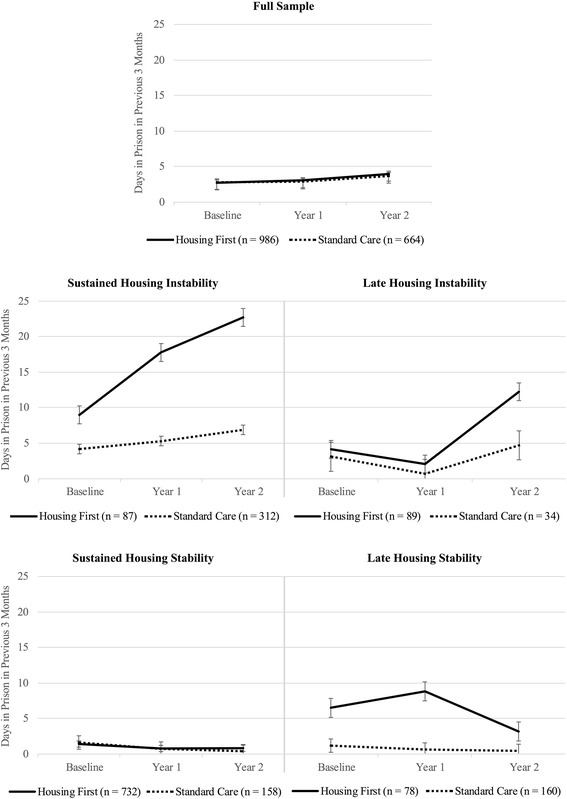
Mean time in prison by intervention and housing stability

A three-way interaction effect between time, intervention, and housing stability was found for days spent in prison in the previous three months (*p* < .001; see Fig. 2). Follow-up linear mixed models conducted separately by intervention condition showed that, for the Housing First group, there was a significant interaction between time and housing stability (*p* < .001). Pairwise comparisons revealed that sustained unstably housed participants had significantly increased time in prison from baseline to 12 months (ASMD = 0.69, *p* < .001, 95% CI = 0.35–1.03), and then again between 12 and 24 months (ASMD = 0.34, *p* = .02, 95% CI = 0.05–0.63). Although the parameter estimates of the complete case analysis for sustained housing instability participants in the Housing First group at 24 months (*M* = 22.72, *SE* = 1.27, 95% CI = 20.24–25.21) moderately diverged from those of the sensitivity analysis (*M* = 20.23, *SE* = 1.38, 95% CI = 17.52–22.94), the magnitude of the effect, as well as the consistency of the statistical findings between the complete case analysis and each individual imputation give confidence in the pattern of findings.

Late unstably housed participants in the Housing First condition also had increased time in prison between 12 and 24 months (ASMD = 0.69, *p* < .001, 95% CI = 0.41–0.98). Overall, the change from baseline to 24 months was a significant increase as well (ASMD = 0.61, *p* < .001, 95% CI = 0.29–0.92). As for late stably housed participants who received Housing First, a significant drop in the amount of prison days was observed between 12 and 24 months (ASMD = 0.35, *p* < .01, 95% CI = − 0.14-0.42). No significant changes in days spent in prison were observed for sustained stably housed participants.

The second linear mixed model, which was conducted with standard care participants, revealed a significant main effect of housing stability (*p* < .001). Pairwise comparisons showed that time in prison among sustained unstably housed participants was significantly higher than those who experienced sustained housing stability (ASMD = 0.27, *p* < .001, 95% CI = 0.16–0.38) or late housing stability (ASMD = 0.28, *p* < .001, 95% CI = 0.18–0.38). No significant temporal changes in prison time were observed for participants in the standard care condition.

## Discussion

Study findings showed that, as homeless people with mental illness became stably housed, their psychiatric hospitalizations decreased, and that use by individuals who experience sustained or late (i.e., recurrent) housing instability remained unchanged. In addition, though visits to the emergency department decreased for all groups, stably housed participants had lower rates overall. As for community services, for sustained and late stably housed participants, their use of food banks increased, whereas their use of homeless shelters and drop-in centers decreased. The changes in health and social service use as homeless people with mental illness become stably housed is further evidence that housing stability can reduce burden on service systems that is caused by higher use of acute services. However, because unstably housed participants also displayed reduced use of emergency departments, homeless shelters, and drop-in centers, housing stability is not a prerequisite for changing use of all health and social services. Nevertheless, supporting tenants to become stably housed should remain the priority of service providers, as this will likely facilitate reduced use of intensive and acute health and social services.

The similarities in health and social service use between the Housing First and standard care conditions in this study suggest that housing stability may be a mediating factor in many of the service use outcomes previously found with the intervention. As previous studies have investigated housing stability and service use as outcomes [18], their interconnectedness has been overlooked. Given that Housing First has been shown to produce superior housing stability outcomes compared to standard care [10–15], this has led to conclusions that the intervention produces the observed service use changes when in actuality it may principally be that people’s housing stability produced by Housing First is responsible for the new patterns of service use. Future studies on the relationship between housing stability and service use in the context of Housing First is needed, as it will have key implications for the minority of homeless people with mental illness who struggle to become stably housed in Housing First. Such research should also investigate how Housing First affects access to services, which was not examined in our study. Given that use of one service can facilitate access to another [35], it is possible that the accompanying support provided to Housing First tenants via ACT or ICM may facilitate timely and appropriate access to other community health and social services. In this way, though housing stability is key to changing service use patterns, Housing First may effect greater change in people’s access to services.

The minimal differences in service use outcomes between Housing First and standard care participants is inconsistent with past research. In particular, studies have found that Housing First is associated with greater use of outpatient resources [20], yet our findings showed that the standard care condition had greater use of hospital-based outpatient services than the Housing First group. This may be due to the specificity of the studied service, as use of ambulatory services at community clinics would not have been accounted for. Moreover, Housing First participants’ use of ACT or ICM support was not examined, and these services may have been primary sources of care for this group. Still, the finding may suggest that any increased use of outpatient services that is observed following entry into a Housing First program may be from use of community-based services rather than hospital-based ones. Examining sources of outpatient service use following Housing First entry is necessary to achieve a better understanding of how the intervention alters use of this type of service. Another notable difference between the intervention groups was in use of drop-in centers. For sustained housing stability participants, use was higher among those that received Housing First than standard care. Given that some people in Housing First models report difficulties with isolation [36], drop-in centers may be an outlet for social connection. A greater understanding of how other community services, such as drop-in centers, can complement Housing First would be particularly beneficial for future program planning and development.

Food banks were one type of service in which participants who became stably housed had increased use. In the context of Housing First, this finding suggests that, although the intervention helps people to exit homelessness, it does not fully resolve the associated consequences of poverty, including food security. Connections to vocational supports and food banks, as well as access to social assistance and disability benefits may be facilitated [37] but people continue to live on low incomes that are insufficient or barely sufficient for getting by. As the scaling up of Housing First continues, consideration should be given to the integration of additional services to address new challenges faced by tenants after homelessness. Given that more than half of Housing First tenants who want to return to work are willing to explore individual placement and support opportunities [38], coupling supported employment with supported housing may further help people with mental illness to exit poverty.

Unlike use of most health and social services, time spent in prison was a domain where the Housing First and standard care groups differed significantly as a result of participants’ housing stability. These findings suggest that any ongoing involvement in the legal system or continued criminal activity may be a key risk factor to achievement of housing stability within the Housing First model. For this reason, prioritization of tenants’ legal needs and provision of support via the ACT or ICM teams may further improve the intervention’s housing stability outcomes. The ACT support model has been modified for use with forensic populations and these adaptations (e.g., having law enforcement and probation officers on the ACT team, recruiting criminal justice sector agencies as partners, targeting recidivism prevention as a primary outcome, having specialized risk assessments) [39, 40] may be particularly helpful in supporting some Housing First tenants to become stably housed. Our findings also suggest that making Housing First services readily available to individuals being discharged from prisons could prevent their homelessness.

### Limitations

Several limitations were present in this study. First, despite the overall large sample size, a small proportion of participants were stably housed in the first 12 months but unstably housed in the last 12 months (i.e., late housing instability), especially within the Housing First condition. As a result, statistical power to identify three-way interactions may have been insufficient. However, as one was found for use of prisons, large effects were still discernable. Second, recruitment did not involve the random selection of participants from those individuals referred to the trial. However, information about the study was disseminated to a wide range of health, social, community, and correctional service agencies in order to obtain a sample that was representative of the adult homeless population in each city where the trial was being conducted. Third, this study did not account for use of services provided via the ACT and ICM teams among Housing First participants. The support teams may have affected use of services, particularly mental health services accessed via hospital settings. Moreover, because Housing First participants were randomized to receive ACT or ICM based on their level of need, the trial design limits further examination of the impacts that the support teams can have during the critical, first two years in housing. Fourth, because service use data was self-reported, the information may not accurately reflect participants’ service use. However, in a subsample of the one used in this study, Somers et al. [32] found that participants reliably reported their overall use of health and justice services, offering confidence in the accuracy of data in this study. Lastly, the two-year study period represents a time of adjustment and settling in for many homeless people with mental illness. The long-term impacts of housing stability on service use remain unknown and require further study.

## Conclusions

Overall, findings show that, as homeless people with mental illness become stably housed, their use of a range of services changes. Moreover, temporal changes were largely similar between the Housing First and standard care groups, suggesting that people’s housing stability is a key factor contributing to many of the observed changes in service use. To reduce homeless people with mental illness’ reliance on emergency and institutional services, the primary, initial objective of mental health housing programs must be to continue to focus on stably housing individuals. Although Housing First is effective at achieving this goal for many people, there is still a minority of individuals who do not succeed in the housing model. One group of people who may be at greater risk of experiencing difficulties achieving housing stability are individuals who have ongoing involvement in the legal system or continued criminal activity. Although modifications to the Housing First support model to better serve people with forensic backgrounds could be beneficial, it is also necessary to consider other housing options that offer high levels of support. To further reduce burden on service systems associated with homelessness and mental illness, more study into whether individuals who experience difficulties with Housing First programs can be stably housed via other housing models is required.

## Abbreviations

ACT: Assertive Community Treatment
ASMD: Adjusted standardized mean difference
GAIN-SPS: Global Appraisal of Individual Needs–Substance Problem Scale
HSJSU: Health, Social, and Justice Service Use Inventory
ICM: Intensive Case Management
MCAS: Multnomah Community Ability Scale
MINI: Mini International Neuropsychiatric Interview
RCT: Randomized controlled trial
RTLFB: Residential Time-line Follow-back
